# Changes in hepatitis C virus antibody titer and viral RNA load in non-Hodgkin's lymphoma patients after rituximab chemotherapy

**DOI:** 10.1111/j.1751-553X.2008.01034.x

**Published:** 2009-08

**Authors:** Y Tsutsumi, K Ichiki, S Shiratori, T Kawamura, J Tanaka, M Asaka, M Imamura, N Masauzi

**Affiliations:** * Department of Internal Medicine, Hakodate Municipal HospitalHakodate; Departments of † Hematology and Oncology, Hokkaido University Graduate School of MedicineSapporo, Japan; ‡ Gastroenterology, Hokkaido University Graduate School of MedicineSapporo, Japan E-mail: yutsutsu@shore.ocn.ne.jp

**Figure 1:**
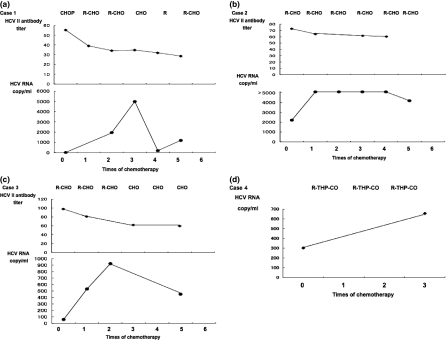
Time course of HCV-RNA load and HCV-antibody titer. (a) HCV-RNA load was elevated after treatment with rituximab or a chemotherapy regimen containing rituximab. (b) HCV-RNA load was elevated and HCV-antibody titer was decreased after rituximab and CHO. (c) HCV-RNA load was elevated after rituximab-containing regimen and decreased after chemotherapy without rituximab. HCV-antibody titer was decreased after rituximab-containing regimen. (d) HCV-RNA load was elevated after rituximab-containing regimen.

*Sir*, We found that four of the 84 B-cell non-Hodgkin’s lymphoma (NHL) patients treated with rituximab in our institution from March 2004 to March 2007 were positive for hepatitis C virus (HCV) antibody. We analyzed the HCV-RNA and HCV antibody levels in these four patients (two male and two female, with ages ranging from 43 to 80) and the characteristics of their cases are listed in Table 1. Each patient had diffuse large B-cell type lymphoma (DLBL) and hepatitis C antibodies (HCV II, this antibody was calculated IgG for HCV). The HCV-RNA load in each patient was measured (in the same laboratory) by using the polymerase chain reaction (PCR) in a method described elsewhere (Takeuchi *et al.*, 1999). Their HCV-RNA loads ranged from 20 to 2100 KIU/ml (more than 5 KIU/ml was positive; max 5000 KIU/ml) in all patients. All cases received rituximab (375 mg/m^2^ on day 1) plus CHOP or a CHOP-like regimen (cyclophosphamide 750 mg/m^2^ on day 2, vincristine 1.4 mg/m^2^ on day 2, doxorubicin 50 mg/m^2^ on day 2, and with/without prednisolone 60 mg/day from day 2 to day 6) or a THP-CO (cyclophosphamide 500 mg/m^2^ on day 2, vincristine 1.0 mg/m^2^ on day 2, pirarubicin 30 mg/m^2^ from day 2 to day 6) regimen at 3-week intervals. One patient received 40-Gy radiotherapy to the bulky residual mass remaining after chemotherapy. They received three to six courses of these regimens (Figure 1). Three patients (cases 2 and 3) obtained a complete response (CR) and one (case 1) obtained an unconfirmed complete response (CRu). The case-4 patient relapsed three years after chemotherapy, but each of the others kept a CR or CRu from one to four years after chemotherapy. The HCV antibodies in all four patients decreased slightly throughout the clinical course. The HCV-RNA load in case 1 was not elevated after the administration of chemotherapy without rituximab (CHOP) but was elevated after the administration of R-CHO (CHOP without predonisolone) and was decreased after the administration of CHO (without rituximab). In this case, the HCV-RNA load was elevated only after the administration of rituximab. In the other cases, the HCV-RNA load also increased gradually elevated after the administration of R-CHO or R-THP-CO (THP-COP without predonisolone). In case 3, the HCV-RNA load increased gradually elevated after the administration of R-CHO and decreased after the administration of CHO. The HCV-RNA load was elevated after the administration of rituximab or a rituximab-containing regimen and was decreased after the treatment without rituximab.

**Table 1:** HCV antibody or HCV-RNA status of the patients with administration of rituximab

Cases	Age/ gender	Disease	HCV-II (before/ after)	Anti HCV-II titer with rutuximab treatment (before/after)	HCV-RNA with rutuximab treatment (before/after)	Ch E (before/ after)	Maximum AST/ALT during treatment	Number of rituximab treatments	Outcome
Case 1	55/M	DLBCL IV	+/+	55.4/28.7	20/5000	141/156	295/230	4	Alive
Case 2	43/F	DLBCL III	+/+	73/60.6	2220/5000<	301/281	65/62	6	Alive
Case 3	74/M	DLBCL IV	+/+	98.2/59.9	62/924	178/116	171/124	3	Alive
Case 4	80/F	DLBCL IV	+/+	+/+	304/657	101/123	101/123	3	Alive

HCV, hepatitis C virus; DLBCL, diffuse large B-cell lymphoma; CS, clinical stage; Ch E, cholinesterase; AST, aspartate aminotransferase; ALT, alanine aminotransferase.

We previously reported changes in the titers of antibodies to HBsAg (anti-HBs) and to hepatitis B core antigen (anti-HBc) that were due to treatment with chemotherapy and rituximab (Tsutsumi *et al.*, 2005). Here, we investigated our HCV-RNA-positive NHL patients after the administration of rituximab and CHO or CHOP. All of these cases showed elevated HCV-RNA load after the administration of chemotherapy and rituximab but showed decreased HCV-RNA load after chemotherapy without rituximab. The HCV-RNA load in case 1 was elevated after the administration of rituximab alone, suggesting that rituximab elevated the HCV viral load. These results suggest that the immune system against HCV may function differently from that against hepatitis B virus.

Some reports showed a correlation between HCV and B-cell type NHL (Mele *et al.*, 2003; de Sanjose *et al.*, 2004; Takeshita *et al.*, 2006). But in these cases, HCV-RNA was not positive for the lymph node specimen involved in the lymphoma. HCV-RNA and the development of NHL were discussed in many cases.

HCV-RNA load elevation after the administration of rituximab and CHOP has been reported by only two groups of investigators (Aksoy *et al.*, 2006; Lake-Bakaar *et al.*, 2007). In those cases, the reactivation of HCV was shown after chemotherapy and rituximab treatment. Lake-Bakaar *et al.* (2007) considered how B-cell depletion affected the reduction of neutralizing IgM for HCV. They reported that anti-E1/E2 HCV antibodies were not affected by B-cell depletion, but we found HCV antibodies to be decreased. The HCV antibody we examined was IgG, and the difference between our results and those reported previously suggests that the IgM response for HCV might not be paralleled by the IgG response. Although B-cell depletion continued for several months, the HCV viral load did not remain elevated, suggesting that the B-cell depletion was not the only reason for the elevated HCV viral load. The number of regulatory T cells, on the other hand, was elevated 30 days after administration of rituximab and decreased within 90 days (Vigna-Perez *et al.*, 2006). The decreased population of T cells may have been too small to suppress the HCV viral load. Although aspartate aminotransferase (AST) and alanine aminotransferase (ALT) levels were elevated in all cases, cholinesterase was decreased in only one. This may show that HCV-RNA load elevation was not the problem of standard chemotherapy and rituximab treatment for lymphoma patients. But in follicular lymphoma, maintenance therapy of the periodical administration of rituximab may lead to the continuous elevation of the HCV-RNA load and consequently may induce liver cirrhosis. We found that rituximab leads to elevated HCV viral load during the clinical course of lymphoma. Periodic administration of rituximab for an extended period of time may lead to the continuous elevation of HCV-RNA load and compromise the liver function of HCV-positive lymphoma patients.
